# Unravelling the Contribution of the rs7041 and rs4588 Polymorphisms of the *GC* Gene and Serum VDBP Levels for Developing Metabolic Syndrome in the Mexican Population

**DOI:** 10.3390/ijms231810581

**Published:** 2022-09-13

**Authors:** Alberto Hidalgo-Bravo, Berenice Rivera-Paredez, Guadalupe León-Reyes, Nelly Patiño, Manuel Castillejos-López, Jorge Salmerón, Rafael Velázquez-Cruz

**Affiliations:** 1 Departamento de Genética, Instituto Nacional de Rehabilitación (INR), Mexico City 014389, Mexico; 2 Centro de Investigación en Políticas, Población y Salud, Facultad de Medicina, Universidad Nacional Autónoma de México (UNAM), Mexico City 04510, Mexico; 3 Laboratorio de Genómica del Metabolismo Óseo, Instituto Nacional de Medicina Genómica (INMEGEN), Mexico City 14610, Mexico; 4 Instituto Nacional de Medicina Genómica (INMEGEN), Mexico City 14610, Mexico; 5 Departamento de Epidemiología, Instituto Nacional de Enfermedades Respiratorias (INER), Mexico City 14080, Mexico

**Keywords:** metabolic syndrome, serum vitamin D binding protein, *GC* gene, single nucleotide polymorphism, haplotype

## Abstract

Metabolic syndrome (MetS) is a multifactorial disorder integrated by a constellation of cardiovascular risk factors. The genetic and environmental determinants of MetS are not fully elucidated. This study investigated the association of two common single nucleotide polymorphisms (SNPs) on *GC*, rs7041 and rs4588, derived haplotypes, and serum vitamin D binding protein (VDBP) levels with the susceptibility to suffer MetS in Mexican adults. We included 1924 individuals; clinical and biochemical data were obtained through standard methods. Genotyping was performed through predesigned TaqMan assays. Logistic regression models were used to assess the associations of interest. Prevalence of MetS was 52.9% in the whole population, being more frequent in women. We observed that some association results differed between sexes. The GG genotype of the rs7041 was associated with increased odds of MetS in women. For the rs4588, the CA genotype had a protective effect against MetS in women. The haplotype GC2 was associated with reduced odds for MetS and some of its components in women. Our data suggest that VDBP serum levels were influenced by genotypes/haplotypes and this interplay seems to influence the risk of MetS. Our data provide reliable evidence regarding the association of *GC* polymorphisms with MetS risk in Mexican women.

## 1. Introduction

Metabolic syndrome (MetS) is a pathological condition defined as a constellation of cardiovascular risk factors: central obesity, elevated fasting plasma glucose (FPG), low high-density lipoprotein cholesterol (HDL-c), and high blood pressure (BP) [1]. The presence of MetS can lead to the development of cardiovascular disease (CVD) and type 2 diabetes mellitus (T2DM) [2]. A recent meta-analysis calculated a global prevalence of MetS between 12.5% and 31.4%, depending on the criteria employed [3]. Based on the Adult Treatment Panel III (ATP III) criteria, the prevalence of MetS in the Mexican population is 41.6%, one of the highest worldwide. MetS prevalence has been continuously growing in the last decades, and the national health and nutrition survey demonstrated a 22% increase from 2006 to 2018. The rising prevalence of MetS was accompanied by a higher risk of suffering CVD [4]. The continuous rising prevalence of MetS in Mexico is mainly attributed to genetic factors, consumption of high-carbohydrate diets, sedentary lifestyles, and insufficient public policies for preventing MetS in the general population [5]. The etiology of MetS and its components involves complex interactions between genetic and environmental factors, such as aging, lifestyle, proinflammatory disorders, and hormonal changes. Heritability of MetS ranges from 13% to 30%, and for some of its components, it is as high as 50% [6].

Based on current evidence, vitamin D (VD) has emerged as a relevant factor in the chain of events involved in MetS occurrence [7]. Several authors have found an inverse association between VD levels and incidence of MetS [8,9]. Furthermore, longitudinal studies have demonstrated that VD supplementation reduces the occurrence of MetS [10]. Different mechanisms have been proposed for supporting the role of VD on MetS development. VD can affect insulin secretion and response to insulin in different peripheral tissues. In addition, it has been proposed that obesity leads to VD deficiency through a sequestering mechanism due to its liposoluble nature, reducing the availability of VD [11]. Regarding dyslipidemia, VD increases calcium intestinal absorption and reduces absorption of lipids. Furthermore, VD inhibits the parathyroid hormone reducing lipogenesis [12]. Vitamin D binding protein (VDBP) is the main transporter of VD in serum [13]. In addition, VDBP can bind to fatty acids and act as a potent actin scavenger [14]. VDBP is encoded by the GC gene, which is recognized for being highly polymorphic. Two common single nucleotide polymorphisms (SNPs), rs7041 and rs4588, on *GC* originate the three main and best characterized haplotypes. The SNP rs7401 is a T/G transversion at codon 432, resulting in the substitution of asparagine for glutamine. The rs4588 SNP is a C/A transversion at codon 436, resulting in the change of threonine for lysine. The haplotypes are identified as follows: GC1F (rs7041-T/rs4588-C; Asp/Lys); GC1S (rs7041-G/rs4588-C; Glu/Thr); and GC2 (rs7041-T/rs4588-A; Asp/Lys) [15]. There is evidence suggesting that these three isoforms impact VDBP concentration and VD availability, which can affect VD functions [11,16]. A few studies on Chinese and Thai populations have explored the relationship between the main *GC* variants and MetS [17,18]. Genetic variants on *GC* have never been investigated in relation to MetS in the Mexican population. Therefore, the aim of this study was to investigate the association of the two common SNPs, rs7041 and rs4588, derived haplotypes, and serum VDBP levels with susceptibility to MetS in a cohort of Mexican adults.

## 2. Results

### 2.1. Characteristics of the Study Sample

We included a total of 1924 individuals with a prevalence of MetS of 52.9% with statistically significant differences by sex (males 45.8% vs. females 55.7%, *p* = 0.0001). Table 1 depicts the distribution of the clinical-demographic characteristics, biochemical measurements, and alleles/genotypes and haplotypes in the study population. Participants with a diagnosis of MetS were significantly older and tend to be less physically active. Although the VD intake was significantly lower only in the general population, the VD serum levels were lower in individuals with MetS in both sexes. We did not observe differences in VDBP levels according to MetS status.

Analysis of allelic and genotype frequencies in the general population did not show a significant difference by MetS status for both variants. When stratified by sex, the TG and GG genotypes of the rs7041 were significantly more frequent in the group of men (*p* = 0.038) and women (*p* = 0.044) with MetS, respectively. Regarding the haplotype analysis, only the homozygous GC1S were more frequent in the group of women with MetS (*p* = 0.034) and the heterozygous GC2/GC1F in the population of MetS (*p* = 0.036). We found significant differences by MetS status in carbohydrate (*p* = 0.019), protein (*p* = 0.0009), total fat (*p* = 0.037), and VD intake (*p* = 0.025) in the general population (Table 1).

### 2.2. Genetic Association Analysis with MetS

In the global population, the GG genotype of the rs7041 was associated with an increased odds of MetS under the co-dominant model (*p* = 0.027), and in agreement with this, under the dominant model, carriers of the G allele also displayed odds increased (*p* = 0.027). When divided by sex, the increased odds were observed only in women with the GG genotype under the co-dominant model (*p* = 0.029, respectively). For the rs4588, the association in the global population was observed under the co-dominant model for the CA genotype (*p* = 0.049), and for the carriers on the A allele under the dominant model (*p* = 0.023); in both cases the association was protective. The association remained in the same direction in the group of women only under the dominant model (*p* = 0.030). Regarding the haplotypes, there was a protective effect of the GC2/GC1F combination (OR = 0.62, *p* = 0.004). The same effect was found in the women (OR = 0.57, *p* = 0.007), plus the homozygous GC2 were associated also with a reduced odds of MetS (OR = 0.42, *p* = 0.025) (Table 2).

The variant rs4588 was associated with waist circumference (WC) and HDL-c. The analysis with WC revealed a protective association for the carriers of the A allele of the rs4588, under the three inheritance models in the total (*P*_co-dom_ = 0.003, OR = 0.36; *P*_dom_ = 0.003, OR = 0.66; *P*_reces_ = 0.010, OR = 0.41) and women’s population (*P*_co-dom_ = 0.0002, OR = 0.20; *P*_dom_ = 0.0001, OR = 0.49; *P*_reces_ = 0.002, OR = 0.26). With respect to HDL-c, we observed a protective association, under the dominant inheritance model, only in the women’s population (OR = 0.73; *P*_dom_ = 0.014). Moreover, the variant rs7041 was associated with HDL-c and triglycerides (TG). The analysis with HDL-c revealed a risk association for the carriers of the G allele of the rs7041, under co-dominant and dominant inheritance models in the total (*P*_co-dom_ = 0.004, OR = 1.50, *P*_dom_ = 0.003, OR = 1.39) and women’s population (*P*_co-dom_ = 0.014, OR = 1.53; *P*_dom_ = 0.006, OR = 1.45). With respect to TG, we observed a risk association, under co-dominant and dominant inheritance models, in the total (*P*_co-dom_ = 0.041, OR = 1.33; *P*_dom_ = 0.016, OR = 1.30) and men’s population (*P*_co-dom_ = 0.042, OR = 1.75; *P*_dom_ = 0.026, OR = 1.60) (Tables S1–S5).

On the other hand, the haplotype 2/1F was associated with HDL-c and TG. In the analysis with HDL-c, we observed a protective association in the total (*p* = 0.015, OR = 0.66) and women’s population (*p* = 0.009, OR = 0.59). With respect to TG, we also observed a protective association in the total (*p* = 0.006, OR = 0.64) and men’s population (*p* = 0.017, OR = 0.47). The haplotype 2/1S was only associated with blood glucose in the women’s population (*p* = 0.047, OR = 0.69). In addition, the diplotype 2/2 was associated with WC, in the total (*p* = 0.032, OR = 0.44) and women’s population (*p* = 0.002, OR = 0.21), while on the other hand, we did not observe association with BP (Tables S1–S5).

### 2.3. Association between VDBP Serum Levels and Metabolic Syndrome

Table 3 shows the characteristics of subjects according to VDBP categories defined by tertiles. The only significant differences between categories were the age in the general population, at the expense of the women’s group, and the higher proportion of women in the highest category. The SNPs analysis considering this stratification showed that the TT genotype of the rs7041 was less frequent in the highest category of VDBP across all groups, while the GG genotype was the most frequent in the same category. In agreement with this observation, the frequency of the T allele has an inverse relationship with the levels of VDBP; on the contrary, the G allele has a direct relationship. For the rs4588, the frequency of the C allele increases along with the VDBP levels, while the A allele decreases. Therefore, the CC genotype was the most frequent in the highest category of VDBP, while the CA/CC genotypes were the least frequent in the same category.

In the general population, the analysis of the haplotypes revealed that homozygous GC1S and the heterozygous GC1S/GC1F were the most frequent haplotypes in the highest category of VDBP. The less frequent haplotypes were the carriers of one GC2 haplotype in combination with GC1S or GC1F. These findings were also observed in the group of women, but not in men (Table 3).

We observed that women in the highest category of VDBP had an increased odds for MetS compared to women in the lowest category (OR = 1.43, 95%CI 1.07–1.92, *p* = 0.017). The components of MetS that showed a significant association were WC (medium category in general population: OR = 1.49, 95%CI 1.07–2.08, and medium category in men: OR = 1.88, 95%CI 1.11–3.19) and TG. The odds of increased levels of TG were observed in the global population in the highest category (OR = 1.80, 95%CI 1.41–2.26, *p* = 1.6 × 10^−6^) and in women in the middle (OR = 1.68, 95%CI 1.27–2.23) and highest category (OR = 2.07, 95%CI 1.56–2.75) (data not shown).

To further explore the relation of VDBP levels to MetS, correlation tests were performed between VDBP and each component of MetS. The results revealed small significant negative correlations of WC and systolic BP with VDBP (*p* = 0.0044 and *p* = 0.0003, respectively). The correlation of systolic BP with VDBP was observed also in men and women (*p* = 0.028 and *p* = 0.016, respectively). On the other hand, the analysis revealed significant positive correlations of HDL-c, and TG with VDBP, in the entire studied population (*p* = 0.0011 and *p* = 0.0044, respectively), and the women’s group (*p* = 0.0015) (Table 4).

## 3. Discussion

MetS represents a major worldwide health issue; however, its multifactorial etiology has not been completely elucidated, and studies analyzing its etiology are scarce. Parsing the nature of MetS is essential for identifying the population at higher risk and designing public policies for preventing MetS in the general population. Herein, we analyzed the effect of VDBP, and its two common variants and respective haplotypes, on MetS and its components. VDBP has a key role on the distribution and bioavailability of VD, and there is evidence showing that VDBP variants can affect serum concentration of VD and, consequently, VD functions [19,20,21]. We observed reduced levels of VD in women and men with MetS. This relationship has been constantly found in populations from all continents (reviewed in [10]). One proposed mechanism for this association is that VD promotes pro-insulin conversion into insulin in pancreatic β-cells. This hypothesis is supported by the VD receptor (VDR) expression in these cells [22].

Our findings provide reliable evidence regarding the association of the *GC* polymorphisms with MetS risk in the Mexican population. When considering the SNP rs7041, it is interesting to notice that genotypes behave differently between women and men. The effect of the SNP rs7041 on serum VDBP levels and metabolic traits has been previously described [19,23]. In the Caucasian population, the presence of the G allele significantly increased the risk of heart failure in combination with low levels of VD in serum [24]. Our data revealed that the G allele is associated with increased odds of MetS under different inheritance models; this effect is mainly observed in women. Furthermore, when looking at MetS components, we observed an association of this allele with an increased odds of low HDL-c in women and elevated TG levels in men.

Regarding the association of the SNP rs4588 with MetS, we observed a protective effect in the presence of the A allele under the co-dominant and dominant inheritance models. This effect is mainly depending on the group of women. Association analysis with individual components of MetS showed a protective effect for the carriers of the A allele against increased WC, mainly derived from the effect on women. There is also a protective effect on low HLD-c of the A allele, exclusively in women. Nevertheless, previous studies have not found an association of alleles or genotypes of the rs4588 SNP with components of MetS [25,26].

The distribution between MetS and non-MetS groups showed that homozygous GC1S are more frequent in women with MetS, and heterozygous GC2/1F are more frequent in the general population with MetS. In addition, the GC2/1S and the GC1/1F haplotypes were protective factors against T2DM and low HDL-c, respectively, in women. In the group of men, the GC1S/1F was associated as a risk factor for increased WC. In addition, also in men, the homozygous GC1F had protection against low HDL-c, and the heterozygous GC2/1F showed a protective effect against hypertriglyceridemia. It can be noticed that the GC2 haplotype is present in most cases when a protective effect is observed.

We did not observe differences of serum VDBP concentrations between MetS status. This result agrees with what has been observed in Polish and Chinese populations [17,27]. In contrast, a study in a population from Thailand reported lower levels of serum VDBP in individuals diagnosed with MetS [18]. These discrepancies may be due to the genetic background of each population, the small sample size of previous studies, and the limited number of studies replicating these findings [28]. Given the larger sample size, the current study may better represent the relationship between VDBP levels and MetS.

To look in more detail at the influence on VDBP variants on its serum levels and MetS, we stratified the study population by serum VDBP categories defined by tertiles. The G allele of the rs7041 was more frequent in the highest VDBP serum category than in the lowest. The rs4588-C allele is more frequent in the highest category of VDBP; in contrast, the A allele was more frequent in the lowest category. Our results differ from previous reports where the A allele has been associated with increased concentrations of VDBP in Caucasian and Afro-American populations in the United States [19]. On the other hand, in the Chinese population, no significant association was found [17].

The distribution of the haplotypes across the VDBP categories defined by tertiles revealed that homozygous GC1S and heterozygous GC1S/1F were more frequent in the highest category. In contrast, GC2/1S or GC2/1F were more frequent in the lowest category. As a factor for developing MetS, the homozygous GC2 and the GC2/1F haplotype showed a protective effect in women. Interestingly these same haplotypes conferred protection against increased WC also in women. These findings suggest an interplay between haplotypes and VDBP levels for the development of MetS or some of its components.

We noticed that the effect of *GC* alleles, genotypes, and haplotypes differ between women and men. Even when an effect was observed in the general population, it could be attributable to one sex group. Plausible explanations behind this different behavior can be related to an interplay between VDBP and sexual hormones. Serum levels of VDBP raise during pregnancy, and this effect has been demonstrated in different populations suggesting that VDBP expression responds to sexual hormones in women. VDBP levels also can increase in the presence of some ovarian tumors and hormone replacement therapy [29]. Further evidence of a relationship between VDBP and sexual hormones came from detection of elevated levels of VDBP in patients with polycystic ovary syndrome and reduced levels in patients with diminished ovarian reserve [30]. On the contrary, male sex hormones do not seem to have an effect on VDBP concentration [15].

VDBP is involved in different biological processes beyond the transport of VD metabolites. VDBP is able to bind to fatty acids, and the association between VDBP with TG and HDL-c has been demonstrated [31]. A recent study found a positive correlation between VDBP serum levels and fat mass index and a negative correlation with HDL-c in overweight and obese women [32]. Nevertheless, these previous studies did not consider the VDBP haplotypes, and our data contribute to this knowledge by analyzing the main variants of VDBP.

Another potential route by which VDBP can be associated with metabolic traits could be related to is its role in macrophage activation. Macrophages play an active role in the development of insulin resistance and lipid dysregulation through the inflammatory response [33]. VDBP is converted into vitamin D binding protein macrophage activator factor (DBP-MAF) after a series of deglycosylation reactions [34]. Experimental evidence supports differential conversion rates among the three major variants of VDBP. The highest conversion rate is observed for the GC1 alleles, while the lowest is for the GC2 alleles [35]. Based on these facts, it is tempting to hypothesize that the association of the GC2 haplotype with a protective effect could be related to a diminished activation of macrophages secondary to a reduced conversion of the GC2 variant into DBP-MAF. This hypothesis needs to be experimentally validated.

The present study has several strengths. First, this study includes a large number of individuals compared to other studies, which makes results more reliable, and furthermore, this cohort has been widely characterized in previous reports. Second, our study is the first to evaluate the relationship between *GC* variants, serum VDBP levels, and MetS risk in the Mexican population. Third, this study contributes evidence about the complex genetic architecture involved in MetS in the Mexican population. Meanwhile, some limitations of the present study should be acknowledged. First, the participants were recruited from the central region of Mexico (Morelos); therefore, additional studies are required to determine if the observed findings can be replicated in individuals from other areas of Mexico. Second, the study was cross-sectional, which did not allow for the determination of causal relationships between *GC* polymorphisms and MetS. These findings should be validated using longitudinal studies. Third, only two SNPs were included in the study, and for a better understanding of the role of all *GC* variants, whole-gene sequencing should be required in future studies.

## 4. Materials and Methods

### 4.1. Study Population

We performed a cross-sectional analysis on the participants of the Health Workers Cohort Study (HWCS), comprising subjects who were followed up between 2010 and 2012. The study design, methodology, and participants’ baseline characteristics have been described in previous reports [36]. Briefly, the HWCS is a prospective cohort study composed of 2085 active and retired health workers, from the Instituto Mexicano del Seguro Social (IMSS), located in Cuernavaca, Morelos, who have a DNA sample. In order to evaluate the association of interest, we excluded individuals < 18 years of age (*n* = 85), outliers of VDBP levels (*n* = 28), and missing WC data (*n* = 15). After these exclusions, a total of 1924 subjects comprised the study sample. HWCS approval was provided by the Institutional Review Board (12CEI 09 006 14), the procedures agreed with the Declaration of Helsinki, and study participants provided informed consent.

### 4.2. Outcome Definition

MetS was defined according to the criteria published by the National Cholesterol Education Program (NCEP)-ATP III: (1) blood glucose ≥100 mg/dL or diagnosed T2DM, (2) low HDL-c < 40 mg/dL in men and < 50 mg/dL in women, (3) triglycerides ≥ 150 mg/dL, (4) BP ≥ 130/85 mmHg, and (5) increased WC ≥ 102 cm in men ≥ 88 cm in women [1].

### 4.3. Genotyping

DNA was isolated from peripheral blood leukocytes using the QIAamp DNA Blood Mini Kit, following the manufacturer’s instructions. The *GC* SNPs rs4588 and rs7041 were genotyped using predesigned TaqMan SNP Genotyping assays (Applied Biosystems, Waltham, MA, USA), in a QuantStudio 7 Flex Real-Time PCR system (Applied Biosystems, Waltham, MA, USA). Automatic variant call was undertaken by the instrument software.

### 4.4. Biochemical, Clinical, and Anthropometric Measures

Venous blood samples were collected after 8 h of fasting. Total serum 25(OH)D was measured through the LIAISON^®^ 25OH Vitamin D Total Assay (Diasorin) (intra- and inter-assay variation coefficients were <10%) [37]. VDBP was measured in serum using the ELISA technique with a commercial kit (Quantikine ELISA kit, R&D Systems, Minneapolis, MN, USA, Cat No. DVDBP0B). This kit employs quantitative sandwich enzyme immunoassay using a monoclonal antibody (intra- and inter-assay coefficient of variation, <7%) [38].

Glucose levels were assessed by the oxidized glucose method. TG levels were determined by the colorimetric method after enzymatic hydrolysis with the lipases technique, and HDL-c was measured by the elimination of chylomicron and subsequent catalase. These biomedical assays were processed using a Selectra XL instrument (Randox, Crumlin, UK) [36].

BP measurement was taken by a nurse with an electronic digital BP monitor using standardized procedures. A structured questionnaire was used to obtain information on age, sex, smoking status, medication use (hypoglycemic medications), physical activity, and diet. Leisure-time physical activity was estimated with a previously validated physical activity questionnaire [39], and Word Health Organization (WHO) criteria were used to classify individuals as active [40]. VD intake was assessed using a semi-quantitative food and beverage consumption frequency questionnaire designed and validated in the Mexican population [41]. VD content was then estimated using nutrient profiles from the software program ‘Sistema de Evaluación de Hábitos Nutricionales y Consumo de Nutrimentos’, which was developed by the National Institute of Public Health of Mexico [42].

WC was measured to the nearest 0.1 cm at the highest point of the iliac crest with the subject standing using a flexible fiberglass measuring tape (Seca). Body weight was measured with a calibrated electronic scale (model BC-533; Tanita) and height was measured using a conventional stadiometer (Seca) [36]. Body mass index (BMI) was determined (calculated as weight (kg)/height (m)^2^) and classified into three groups: normal < 25 kg/m^2^; overweight 25–29.9 kg/m^2^, and obesity ≥ 30 kg/m^2^.

### 4.5. Statistical Analyses

Sociodemographic characteristics were summarized by MetS status and sex. Categorical variables were presented as relative frequencies and continuous variables as median (25–75 percentile). The Wilcoxon rank-sum test was used to compare the medians of the continuous variables and the Chi square test was used to compare proportions of categorical variables. To evaluate the association of genetic variants of the *GC* gene and MetS and its components, we performed a logistic regression model adjusted by age, sex, VD intake, smoking status, VD deficiency, and menopausal status (women only). In the models of the components of MetS, such as elevated glucose, BP, and lipids, we added the BMI as an adjustment variable. For both SNPs, we estimated the inheritance models (co-dominant, dominant, and recessive). In the haplotype model, we considered homozygous GC1S individuals as the reference.

Additionally, the study sample characteristics across categories of VDBP were described as medians with interquartile ranges (IQR) or percentages for categorical variables. To evaluate the differences between the lowest vs. the highest category, Dunn’s test or frequency difference, as appropriate, was used. We explored the association of VDBP categories defined by tertiles with MetS and its components, using a logistic regression model, adjusted by age, sex, VD intake, smoking status, VD deficiency, and menopausal status (women only). In the models of the components of MetS, such as elevated glucose, BP, and lipids, we added the BMI as an adjustment variable. A two-sided *p*-value < 0.05 was regarded as the threshold for statistical significance. Finally, we performed Spearman’s correlation between VDBP levels and MetS components. Analyses were performed using STATA software, version 14.0.

## 5. Conclusions

In conclusion, our data support an effect of specific gene variants of *GC*, and their respective haplotypes, on the development of MetS and its components in the Mexican population; this effect seems to be sex-dependent. Nevertheless, these results need to be confirmed whether these associations have ethnic specificity. In the future, serum VDBP concentrations should be considered alongside VD levels and *GC* polymorphisms in research on MetS in the Mexican population.

There are different possible routes for achieving this biological effect as postulated above. These are the effect of estrogens, the ability for binding fatty acids and their role as macrophage activators, and their potential participation in glucose control.

## Figures and Tables

**Table 1:** Distribution of sociodemographic characteristics and lifestyle factors according to metabolic syndrome status and sex.

	Total		Men		Women	
	Without MetS	MetS	Without MetS	MetS	Without MetS	MetS
	*n* = 907	*n* = 1017	*n* = 317	*n* = 268	*n* = 590	*n* = 749
Ages, (years) ^a^	45 (33–56)	56 (48–65) *	43 (33–54)	51 (42–59) *	47 (33–57)	57 (50–66) *
Women, %	65.1	73.7 *	-	-	-	-
Physical activity, %						
Active	38.8	31.4 *	44.2	39.2	35.9	28.6 *
Smoking status, %						
Past, %	27.7	27.6	37.5	41.4	22.4	22.7
Current, %	12.7	12.1	21.1	20.5	8.1	9.1
Vitamin D intake (UI/day) ^a^	152.7 (86.9–259.3)	137.0 * (78.3–184.3)	154.3 (83.2–264.5)	129.1 (70.9–246.3)	152.2 (89.5–255.7)	139.6 (82.0–242.4)
Vitamin D levels (ng/mL) ^a^	22.3 (18–26.6)	20.5 * (16.4–24.4)	23 (19–28.3)	21.3 * (17.1–25.7)	21.9 (17.3–25.9)	20.2 * (16.3–23.9)
VDBP (µmol/L) ^a^	270.2 (233.3–316)	272.2 (229.2–316.1)	267.2 (231.8–304.5)	261.7 (223.6–303.6)	272.2 (235.9–318.3)	276.7 (232.1–318.8)
rs7041, %						
TT	29.3	26.0	28.7	22.4	29.5	27.2
TG	49.3	49.9	48.9	57.5 *	49.6	47.1
GG	21.4	24.2	22.4	20.2	20.9	25.6 *
T	53.9	50.9	53.2	51.1	54.3	50.8
G	46.1	49.1	46.9	48.9	45.7	49.2
rs4588, %						
CC	60.0	63.2	62.5	63.4	58.7	63.2
CA	36.8	34.2	34.7	34.0	37.9	34.3
AA	3.2	2.6	2.8	2.6	3.4	2.5
C	78.4	80.0	79.8	80.4	77.7	80.3
A	21.6	19.7	20.2	19.6	22.3	19.7
Diplotype, %						
1S/1S	20.9	23.6	22.2	19.9	20.1	25.0 *
1S/1F	29.7	29.6	31.1	36.3	28.9	27.1
1F/1F	10.0	10.6	9.5	7.5	10.3	11.7
2/2	3.0	2.5	2.5	2.6	3.3	2.4
2/1F	16.5	13.1 *	16.8	12.4	16.3	13.4
2/1S	20.0	20.6	17.8	21.4	21.1	20.4
Diet						
Energy, (kcal/day) ^a^	1809 (1396–2392)	1687 * (1237–2231)	2026 (1554–2591)	1852 * (1393–2504)	1733 (1336–2319)	1630 * (1198–2125)
Carbohydrate, (% energy) ^a^	65.3 (59.2–70.7)	66.4 * (60.3–71.9)	64.0 (58.5–70.1)	65.1 (57.5–70.7)	66.3 (59.7–71.0)	66.9 * (61.3–72.6)
Protein, (% energy) ^a^	12.3 (10.6–14.0)	12.6 * (11.0–14.4)	12.3 (10.5–13.9)	12.4 (10.8–14.3)	12.3 (10.6–14.1)	12.7 * (11.2–14.4)
Total fat, (% energy) ^a^	21.1 (17.3–21.5)	20.5 * (17.0–24.3)	20.8 (16.9–24.8)	20.4 (16.7–25.2)	21.3 (17.5–25.3)	20.5 * (17.3–24.1)
Vitamin D intake (UI/day) ^a^	152.7 (86.9–259.3)	137.0 * (78.3–243.0)	154.3 (83.2–2645)	129.1 (70.9–246.3)	152.2 (89.5–255.7)	139.6 (82.0–242.4)

^a^ Median (P25–P75). * *p* value < 0.05.

**Table 2:** Association of GC variants with metabolic syndrome stratified by sex.

	Total		Men		Women	
	OR (95%CI)	*p* Value	OR (95%CI)	*p* Value	OR (95%CI)	*p* Value
**rs7041**						
TT	Ref.		Ref.		Ref.	
TG	1.24 (0.98–1.57)	0.069	1.52 (0.99–2.30)	0.051	1.15 (0.87–1.53)	0.332
GG	1.37 (1.04–1.80)	0.027	1.23 (0.73–2.05)	0.435	1.45 (1.04–2.02)	0.029
TT	Ref.		Ref.		Ref.	
TG + GG	1.27 (1.03–1.59)	0.027	1.43 (0.96–2.13)	0.080	1.24 (0.95–1.62)	0.106
TT + TG	Ref.		Ref.		Ref.	
GG	1.18 (0.94–1.50)	0.144	0.92 (0.60–1.41)	0.707	1.33 (1.00–1.76)	0.051
**rs4588**						
CC	Ref.		Ref.		Ref.	
CA	0.81 (0.66–0.99)	0.049	0.88 (0.61–1.27)	0.480	0.79 (0.61–1.01)	0.064
AA	0.57 (0.31–1.00)	0.052	0.67 (0.23–1.96)	0.465	0.50 (0.25–1.02)	0.057
CC	Ref.		Ref.		Ref.	
CA + AA	0.79 (0.65–0.97)	0.023	0.86 (0.60–1.23)	0.411	0.76 (0.60–0.97)	0.030
CC + CA	Ref.		Ref.		Ref.	
AA	0.60 (0.34–1.08)	0.090	0.71 (0.24–2.05)	0.522	0.55 (0.27–1.11)	0.096
**Diplotype**						
1S/1S	Ref.		Ref.		Ref.	
1S/1F	0.90 (0.69–1.19)	0.471	1.28 (0.79–2.07)	0.319	0.77 (0.55–1.08)	0.135
1F/1F	0.98 (0.68–1.42)	0.923	0.98 (0.48–2.00)	0.952	0.95 (0.61–1.47)	0.807
2/2	0.55 (0.29–1.03)	0.062	0.95 (0.30–2.96)	0.929	0.42 (0.20–0.90)	0.025
2/1F	0.62 (0.44–0.86)	0.004	0.72 (0.40–1.31)	0.285	0.57 (0.38–0.86)	0.007
2/1S	0.90 (0.69–1.22)	0.514	1.24 (0.72–2.14)	0.435	0.79 (0.55–1.14)	0.215

Models adjusted for age, sex, VD intake, smoking status, VD deficiency, and menopausal status (women only).

**Table 3:** Distribution of sociodemographic characteristics and lifestyle factors according to VDBP categories defined by tertiles.

	Total			Men			Women		
	VDBP (µmol/L)			VDBP (µmol/L)			VDBP (µmol/L)		
	Low	Medium	High	Low	Medium	High	Low	Medium	High
	*n* = 642	*n* = 641	*n* = 641	*n* = 195	*n* = 195	*n* = 195	*n* = 447	*n* = 446	*n* = 446
	<246.3	246.3–<299.5	>299.5	<241.2	241.2–<289.7	>289.7	<246.4	246.4–<304	>304
Ages, (years) ^a^	53 (43–64)	50 (39–61)	52 * (40–61)	48 (37–58)	44 (36–55)	46 (35–56)	55 (46–66)	53 (42–63)	53 * (41–61)
BMI (Kg/m^2^) ^a^	26.6 (24.1–29.7)	26.9 (24.2–30)	26.7 (23.7–29.6)	26.5 (24.2–29.1)	27 (24.7–29.4)	26.3 (23.6–28.8)	26.8 (24.1–29.9)	26.9 (24–30.3)	26.9 (23.9–30.1)
Women, %	65.7	67.9	75.2 *	-	-	-	-	-	-
Physical activity, %									
Active	35.1	35.7	33.9	39.5	44.6	41.5	32.2	31.2	32.1
Smoking status, %									
Past, %	27.6	27.9	27.5	38	38	42	21.5	22.2	24
Current, %	14.0	11.5	11.5	21	21	20.5	10.3	7.2	8.5
VD intake (UI/day) ^a^	144.6 (84–252.7)	147.3 (85.3–260.4)	137.2 (80.7–239.4)	141.6 (70.1–271)	147.3 (75.8–261.3)	145.2 (78.8–243.7)	149.6 (89.9–246)	146.9 (87.1–255.9)	133.8 (81–240)
VD levels (ng/mL) ^a^	20.9 (17.1–24.7)	21.7 (17.7–25.8)	21 (16.8–25.6)	22.3 (18.5–26.9)	22.2 (18.5–27.2)	21.8 (17.6–27.4)	20.4 (16.3–23.8)	21.3 (17.2–25.4)	20.8 (16.6–24.9)
MetS, %	53.6	51.0	54.0	49.2	42.6	45.6	55.0	56.1	56.7
WC (cm) ^a^	94 (87–102)	94 (87–102)	93 (85–99) *	97 (90–104)	96 (91–102)	95 (88–101)	93 (85–101)	92 (85–101)	92 (85–99)
Systolic BP, (mmHg) ^a^	119 (198–131)	118 (108–129)	116 (106–128) *	122 (113–132)	121 (114–130)	120 (111–131)	117 (107–131)	117 (106–128)	114 (105–127) *
Diastolic BP, (mmHg) ^a^	74 (67–81)	74 (68–81)	74 (68–80)	76 (70–83)	78 (71–84)	77 (69–85)	72 (65–79)	73 (66–80)	73 (67–79)
FPG (mg/dL) ^a^	97 (91–106)	96 (90–105)	97 (90–106)	98 (91–107)	98 (92–106)	99 (92–109)	97 (91–105)	95 (89–104)	97 (90–105)
Triglycerides (mg/dL) ^a^	147 (108–203)	154 (109–210)	163 (119–212) *	167 (116–241)	169 (113–244)	169 (126–256)	141 (105–189)	153 (109–204)	163 (117–207) *
HDL-c (mg/dL) ^a^	43 (36–51)	44 (37.51)	45 (39–53) *	39 (34–45)	40 (34–45)	40 (35–48) *	45 (39–54)	46 (39–54)	46 (40–54) *
rs7041									
TT	32.7	27.6	22.2 *	33.3	23.1	21 *	32.9	29	22.9 *
TG	46.7	50.3	51.8	47.2	55.9	55.4	46.1	49.4	49.1
GG	20.6	22	26.0 *	19.5	21	23.6	21.0	21.6	28.0 *
T	56.1	52.8	48.1*	56.9	51	48.7 *	55.9	53.7	47.4 *
G	43.9	47.2	51.9 *	43.1	49	51.3 *	44.1	46.3	52.6 *
rs4588									
CC	52.3	64.7	68.1 *	55.4	65.1	68.2 *	51.2	63.2	69.2 *
CA	43.3	33.2	29.7 *	40.5	32.8	29.7 *	44.5	34.1	23 *
AA	4.4	2.0	2.2 *	4.1	2.1	2.1	4.3	2.7	1.8 *
C	74	81	83 *	75.6	82	83 *	73.4	80.3	83.7 *
A	26	18	17 *	24.4	18	17 *	26.5	19.7	16.3 *
Diplotype, %									
1S/1S	20	21.3	25.6 *	19	20.7	23.6	20.4	20.7	27.4 *
1S/1F	23.7	31.6	33.5 *	27.8	36.3	36.4	22	29.3	32.4 *
1F/1F	9.1	12.5	9.4	8.7	8.8	8.2	9.5	13.9	10
2/2	4.1	1.9	2.2	4.1	1.6	2.1	3.9	2.7	1.8
2/1F	19.8	13.6	10.7 *	20.6	13	10.8	19.9	12.7	11.3 *
2/1S	23.3	19.1	18.6 *	19.6	19.7	19	24.4	20.7	17 *

^a^ Median (P25–P75) * *p* value < 0.05. *p* value represents the comparison of tertile 3 vs. tertile 1. Abbreviations: blood pressure (BP); body mass index (BMI); fasting plasma glucose (FPG); metabolic syndrome (MetS); vitamin D (VD); waist circumference (WC).

**Table 4:** Spearman correlations between VDBP and MetS components.

	VDBP Levels					
	Total		Men		Women	
Variable	Rho	*p* Value	Rho	*p* Value	Rho	*p* Value
Age	−0.088	0.0001	−0.043	0.3036	−0.129	<0.001
Glucose	−0.012	0.6068	0.019	0.6401	−0.015	0.5948
Waist circumference	−0.065	0.0044	−0.074	0.0744	−0.049	0.0706
HDL-c	0.075	0.0011	0.074	0.0737	0.047	0.0875
Triglycerides	0.065	0.0044	0.044	0.2891	0.087	0.0015
Systolic BP	−0.082	0.0003	−0.090	0.0288	−0.065	0.0167
Diastolic BP	−0.021	0.3585	−0.018	0.6718	0.005	0.8533

Spearman correlation.

## Data Availability

The datasets analyzed in this study are available from the corresponding author on reasonable request.

## Supplementary Materials

The following supporting information can be downloaded at: https://www.mdpi.com/article/10.3390/ijms231810581/s1.
