# Supplementary material for: Unravelling the Contribution of the rs7041 and rs4588 Polymorphisms of the GC Gene and Serum VDBP Levels for Developing Metabolic Syndrome in the Mexican Population
Source: Int J Mol Sci. 2022 Sep 13;23(18):10581. doi: 10.3390/ijms231810581 (PMC9503453; doi:10.3390/ijms231810581)
Supplement: Supplementary file 1 [file ijms-23-10581-s001.zip › ijms-1882240-SI.pdf]

Table S1. Association of GC gene variants with abdominal obesity by sex.

	Total		Men		Women	
	**OR (95% CI)**	***P* value**	**OR (95% CI)**	***P* value**	**OR (95% CI)**	***P* value**
**rs7041**						
TT	Ref.		Ref.		Ref.	
TG	1.23(0.90-1.69)	0.190	1.36(0.84-2.20)	0.207	1.23(0.81-1.87)	0.328
GG	1.12(0.77-1.62)	0.561	0.81(0.46-1.41)	0.452	1.57(0.93-2.66)	0.091
TT	Ref.		Ref.		Ref.	
GG+GG	1.20(0.89-1.60)	0.234	1.16(0.74-1.81)	0.523	1.33(0.89-1.97)	0.161
TT+TG	Ref.		Ref.		Ref.	
GG	0.98(0.71-1.34)	0.887	0.65(0.41-1.05)	0.079	1.38(0.87-2.17)	0.170
**rs4588**						
CC	Ref.		Ref.		Ref.	
CA	0.70(0.52-0.92)	0.012	0.95(0.62-1.45)	0.807	0.53(0.36-0.77)	0.001
AA	0.36(0.18-0.71)	0.003	0.73(0.22-2.42)	0.607	0.20(0.08-0.47)	0.0002
CC	Ref.		Ref.		Ref.	
CA+AA	0.66(0.50-0.87)	0.003	0.93(0.61-1.41)	0.737	0.49(0.34-0.70)	0.0001
CC+CA	Ref.		Ref.		Ref.	
AA	0.41(0.21-0.81)	0.010	0.75(0.23-2.44)	0.628	0.26(0.11-0.60)	0.002
**Diplotype**						
1S/1S	Ref.		Ref.		Ref.	
1S/1F	1.30(0.88-1.91)	0.185	1.82(1.05-3.16)	0.034	1.00(0.58-1.75)	0.986
1F/1F	1.42(0.82-2.43)	0.208	1.26(0.58-2.73)	0.560	1.41(0.64-3.11)	0.390
2/2	0.44(0.21-0.93)	0.032	0.98(0.28-3.44)	0.978	0.21(0.08-0.56)	0.002
2/1F	0.77(0.50-1.19)	0.238	1.24(0.64-2.37)	0.527	0.49(0.27-0.90)	0.021
2/1S	0.87(0.58-1.31)	0.509	1.42(0.77-2.63)	0.262	0.58(0.33-1.02)	0.058

Models adjusted for age, sex, vitamin D intake, smoking status, vitamin D deficiency and menopausal status (only women).

Table S2. Association of GC gene variants with reduced HDL-cholesterol levels by sex.

	Total		Men		Women	
	OR (95% CI)	*P* value	OR (95% CI)	*P* value	OR (95% CI)	*P* value
**rs7041**						
TT	Ref.		Ref.		Ref.	
TG	1.34(1.07-1.69)	0.012	1.20(0.80-1.81)	0.373	1.41(1.07-1.86)	0.016
GG	1.50(1.13-1.97)	0.004	1.40(0.85-2.30)	0.192	1.53(1.09-2.13)	0.014
TT	Ref.		Ref.		Ref.	
GG+GG	1.39(1.12-1.72)	0.003	1.25(0.85-1.85)	0.251	1.45(1.11-1.88)	0.006
TT+TG	Ref.		Ref.		Ref.	
GG	1.24(0.98-1.57)	0.074	1.23(0.81-1.88)	0.329	1.23(0.92-1.65)	0.156
**rs4588**						
CC	Ref.		Ref.		Ref.	
CA	0.85(0.69-1.05)	0.124	1.18(0.82-1.70)	0.375	0.73(0.57-0.94)	0.015
AA	0.76(0.43-1.36)	0.359	0.71(0.25-2.04)	0.531	0.77(0.38-1.54)	0.456
CC	Ref.		Ref.		Ref.	
CA+AA	0.84(0.69-1.03)	0.098	1.14(0.80-1.63)	0.474	0.73(0.57-0.94)	0.014
CC+CA	Ref.		Ref.		Ref.	
AA	0.81(0.46-1.44)	0.478	0.67(0.24-1.88)	0.448	0.87(0.44-1.73)	0.691
**Diplotype**						
1S/1S	Ref.		Ref.		Ref.	
1S/1F	0.93(0.70-1.22)	0.588	0.75(0.47-1.22)	0.247	1.06(0.75-1.51)	0.745
1F/1F	0.65(0.45-0.94)	0.023	0.46(0.23-0.93)	0.030	0.76(0.49-1.19)	0.232
2/2	0.68(0.37-1.25)	0.210	0.65(0.21-2.01)	0.459	0.69(0.33-1.44)	0.321
2/1F	0.66(0.48-0.92)	0.015	0.87(0.49-1.56)	0.642	0.59(0.40-0.88)	0.009
2/1S	0.85(0.62-1.15)	0.282	1.00(0.58-1.72)	0.988	0.80(0.55-1.16)	0.234

Models adjusted for age, sex, VD intake, smoking status, body mass index categories, vitamin D deficiency and menopausal status (only women).

Table S3. Association of GC gene variants with elevated triglyceride levels by sex.

	Total		Men		Women	
	OR (95% CI)	*P* value	OR (95% CI)	*P* value	OR (95% CI)	*P* value
**rs7041**						
TT	Ref.		Ref.		Ref.	
TG	1.29(1.03-1.62)	0.027	1.55(1.00-2.40)	0.049	1.17(0.90-1.54)	0.242
GG	1.33(1.01-1.74)	0.041	1.75(1.02-3.00)	0.042	1.20(0.87-1.64)	0.265
TT	Ref.		Ref.		Ref.	
TG+GG	1.30(1.05-1.61)	0.016	1.60(1.06-2.43)	0.026	1.18(0.92-1.52)	0.194
TT+TG	Ref.		Ref.		Ref.	
GG	1.12(0.90-1.41)	0.313	1.30(0.83-2.04)	0.255	1.08(0.83-1.41)	0.564
**rs4588**						
CC	Ref.		Ref.		Ref.	
CA	0.87(0.71-1.06)	0.163	0.78(0.53-1.15)	0.213	0.91(0.72-1.16)	0.445
AA	0.84(0.47-1.50)	0.548	0.76(0.24-2.36)	0.631	0.87(0.44-1.71)	0.679
CC	Ref.		Ref.		Ref.	
CA+AA	0.86(0.71-1.05)	0.147	0.78(0.53-1.14)	0.200	0.91(0.72-1.15)	0.416
CC+CA	Ref.		Ref.		Ref.	
AA	0.88(0.50-1.57)	0.677	0.84(0.27-2.57)	0.754	0.90(0.46-1.76)	0.755
**Diplotype**						
1S/1S	Ref.		Ref.		Ref.	
1S/1F	0.97(0.74-1.27)	0.835	0.88(0.52-1.47)	0.620	0.97(0.70-1.33)	0.841
1F/1F	0.96(0.67-1.39)	0.842	0.69(0.33-1.46)	0.336	1.09(0.71-1.65)	0.698
2/2	0.88(0.47-1.63)	0.682	0.87(0.25-3.01)	0.830	0.89(0.42-1.78)	0.698
2/1F	0.64(0.46-0.88)	0.006	0.47(0.25-0.88)	0.017	0.71(0.49-1.04)	0.083
2/1S	1.02(0.76-1.37)	0.904	0.93(0.52-1.68)	0.823	1.04(0.74-1.48)	0.811

Models adjusted for age, sex, vitamin D intake, smoking status, body mass index categories, vitamin D deficiency and menopausal status (only women).

Table S4. Association of GC gene variants with elevated fasting plasma glucose (≥100 mg/dL) or previously diagnosed type 2 diabetes by sex.

	Total		Men		Women	
	**OR (95% CI)**	***P* value**	**OR (95% CI)**	***P* value**	**OR (95% CI)**	***P* value**
**rs7041**						
TT	Ref.		Ref.		Ref.	
TG	0.98(0.78-1.24)	0.887	0.91(0.60-1.39)	0.662	1.02 (0.77-1.36)	0.872
GG	1.09(0.83-1.44)	0.524	0.84(0.50-1.40)	0.493	1.24(0.89-1.72)	0.211
TT	Ref.		Ref.		Ref.	
GG+GG	1.02(0.82-1.27)	0.879	0.89(0.60-1.33)	0.564	1.09(0.84-1.42)	0.522
TT+TG	Ref.		Ref.		Ref.	
GG	1.11(0.88-1.40)	0.395	0.89(0.58-1.36)	0.594	1.22(0.92-1.61)	0.168
**rs4588**						
CC	Ref.		Ref.		Ref.	
CA	0.86(0.70-1.06)	0.158	1.03(0.71-1.49)	0.890	0.78(0.61-1.01)	0.059
AA	1.02(0.56-1.83)	0.953	1.09(0.37-3.21)	0.875	0.96(0.47-1.95)	0.916
CC	Ref.		Ref.		Ref.	
CA+AA	0.87(0.71-1.07)	0.185	1.03(0.72-1.48)	0.870	0.79(0.61-1.02)	0.070
CC+CA	Ref.		Ref.		Ref.	
AA	1.08(0.60-1.93)	0.798	1.08(0.37-3.14)	0.889	1.06(0.53-2.13)	0.874
**Diplotype**						
1S/1S	Ref.		Ref.		Ref.	
1S/1F	0.93(0.71-1.23)	0.616	1.00(0.61-1.63)	0.999	0.93(0.66-1.30)	0.665
1F/1F	1.02(0.70-1.47)	0.934	1.95(0.94-4.05)	0.074	0.80(0.51-1.24)	0.312
2/2	0.95(0.51-1.77)	0.867	1.13(0.36-3.58)	0.835	0.87(0.41-1.85)	0.724
2/1F	0.83(0.60-1.16)	0.273	0.95(0.53-1.73)	0.873	0.78(0.52-1.17)	0.231
2/1S	0.84(0.62-1.13)	0.246	1.27(0.73-2.21)	0.396	0.69(0.48-1.00)	0.047

Models adjusted for age, sex, vitamin D intake, smoking status, body mass index categories, vitamin D deficiency and menopausal status (only women).

Table S5. Association of GC gene variants with elevated blood pressure or taking medication for hypertension by sex.

	Total		Men		Women	
	**OR (95% CI)**	***P* value**	**OR (95% CI)**	***P* value**	**OR (95% CI)**	***P* value**
**rs7041**						
TT	Ref.		Ref.		Ref.	
TG	0.93(0.73-1.20)	0.579	0.95(0.61-1.47)	0.811	0.94(0.69-1.28)	0.683
GG	0.99 (0.74-1.34)	0.987	0.93(0.55-1.59)	0.800	1.04(0.72-1.48)	0.844
TT	Ref.		Ref.		Ref.	
GG+GG	0.95(0.75-1.20)	0.684	0.94(0.62-1.43)	0.786	0.97(0.73-1.30)	0.838
TT+TG	Ref.		Ref.		Ref.	
GG	1.04(0.81-1.34)	0.733	0.97(0.62-1.50)	0.885	1.08(0.80-1.46)	0.622
**rs4588**						
CC	Ref.		Ref.		Ref.	
CA	1.06(0.84-1.32)	0.622	1.15(0.78-1.70)	0.475	1.02(0.78-1.35)	0.864
AA	1.06(0.60-1.99)	0.849	0.80(0.26-2.53)	0.715	1.24(0.58-2.68)	0.578
CC	Ref.		Ref.		Ref.	
CA+AA	1.06(0.85-1.31)	0.611	1.12(0.77-1.64)	0.546	1.04(0.79-1.36)	0.777
CC+CA	Ref.		Ref.		Ref.	
AA	1.04(0.56-1.93)	0.899	0.76(0.25-2.36)	0.638	1.23(0.58-2.64)	0.590
**Diplotype**						
1S/1S	Ref.		Ref.		Ref.	
1S/1F	0.88(0.66-1.19)	0.409	1.02(0.62-1.69)	0.932	0.81(0.56-1.17)	0.272
1F/1F	0.87(0.59-1.3)	0.510	0.84(0.39-1.81)	0.663	0.86(0.53-1.37)	0.519
2/2	0.93(0.48-1.81)	0.838	0.74(0.22-2.51)	0.627	1.04(0.46-2.35)	0.920
2/1F	1.06(0.75-1.51)	0.735	1.30(0.71-2.40)	0.395	0.95(0.61-1.47)	0.817
2/1S	0.95(0.69-1.31)	0.738	1.02(0.57-1.80)	0.952	0.93(0.63-1.38)	0.716

Models adjusted for age, sex, vitamin D intake, smoking status, body mass index categories, vitamin D deficiency and menopausal status (only women).
